# Studying Language Change Using Price Equation and Pólya-urn Dynamics

**DOI:** 10.1371/journal.pone.0033171

**Published:** 2012-03-12

**Authors:** Tao Gong, Lan Shuai, Mónica Tamariz, Gerhard Jäger

**Affiliations:** 1 Department of Linguistics, University of Hong Kong, Hong Kong; 2 Department of Chinese, Translation and Linguistics, City University of Hong Kong, Hong Kong; 3 Department of Linguistics and English Language, University of Edinburgh, Edinburgh, United Kingdom; 4 Department of Linguistics, University of Tübingen, Tübingen, Germany; Universita' del Piemonte Orientale, Italy

## Abstract

Language change takes place primarily via diffusion of linguistic variants in a population of individuals. Identifying selective pressures on this process is important not only to construe and predict changes, but also to inform theories of evolutionary dynamics of socio-cultural factors. In this paper, we advocate the Price equation from evolutionary biology and the Pólya-urn dynamics from contagion studies as efficient ways to discover selective pressures. Using the Price equation to process the simulation results of a computer model that follows the Pólya-urn dynamics, we analyze theoretically a variety of factors that could affect language change, including variant prestige, transmission error, individual influence and preference, and social structure. Among these factors, variant prestige is identified as the sole selective pressure, whereas others help modulate the degree of diffusion only if variant prestige is involved. This multidisciplinary study discerns the primary and complementary roles of linguistic, individual learning, and socio-cultural factors in language change, and offers insight into empirical studies of language change.

## Introduction

Language is a dynamic complex adaptive system [1] that undergoes constant changes [2]. Well-documented examples of language change include: the Great Vowel Shift in English during the 14th to 16th century [3], the phonological mergers in Sinitic languages [4], the lexical borrowing among languages [5], [6], and so on. Many changes were achieved via *variant diffusion* (shift in proportions of different variants used by a population of individuals over time [7], henceforth “diffusion”). Regarding the numerous diffusion cases, linguists are curious about the general ways in which diffusion takes place and the separate or collective effects of various factors on this process, with the purpose of identifying *selective pressures on diffusion* (factors that explicitly and consistently drive the diffusion of particular variants in a population) and gaining insights on the human cognitive capacity for language [8]–[12].

Mathematical analysis and computer simulation have recently joined the endeavor to study questions of language evolution. By quantifying contact patterns and constraints within or across populations, mathematical analysis helps predict the outcome of language competition [13]–[18]; by simulating individual behaviors during linguistic interactions, computer modeling helps trace: how local interactions among individuals spur the origin of a common set of lexical items [19], [20], how processing constraints lead to linguistic regularities [21], [22], and how social connections affect diffusion [23], [24].

As for diffusion in particular, the simulation approach usually defines two types of variants (*changed* (*C*) and *unchanged* (*U*) *forms*) and relevant rules to select *C* or *U*. As in [23], [24], individuals are situated in social networks, and choose their forms based on the forms their *neighbors* (individuals directly connected to them) use and the functional bias between *C* and *U*. By repetitively updating individuals' forms and calculating the proportions of *C* and *U* in the population, these studies evaluate *the threshold problem* (minimum bias for *C* to diffuse in the whole population [23]) and the effect of social structures on diffusion. Meanwhile, the mathematical approach usually treats diffusion as a Markov chain, and defines differential equations describing changes among different language states. As in [13], two states, *X* and *Y*, are defined. Change in the proportion of the population using *X* is defined in (1), where *x* and *y* are proportions of individuals respectively using *X* and *Y*, *P_yx_*(*x*,*s*) is the probability of converting from *Y* to *X*, and *P_xy_*(*x*,*s*) is the probability of a reverse conversion:
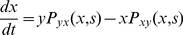
(1)Here, *P_yx_*(*x,s*) = *cx^a^s*, *P_xy_*(*x*,*s*) = *c*(1-*x*)*^a^*(1-*s*), and *c*, *s* and *a* define the attractiveness of *X* or *Y*. Change in the proportion of the population using *Y* can be defined similarly. Analysis on these equations can reveal some stable states of the system. The later work [16] extends [13] by including a bilingual state (*Z*) and redefining the transition equations.

Both of these approaches bear some limitations. On the one hand, simulations are sensitive to initial conditions; without support from mathematical analysis, simulations only offer qualitative understanding. Combining simulation with mathematical analysis can efficiently overcome this limitation. As in [25], the authors unify the two sets of equations in [13] and [16] with agent-based simulations, and discover that individuals' willingness to change languages is prominent for diffusion of a more attractive language and bilingualism accelerates the disappearance of one of the competing languages. On the other hand, Markov models usually involve many parameters and face a “data scarcity” problem (how to effectively estimate the parameter values based upon insufficient empirical data). In addition, the number of parameters increases exponentially with the increase in the number of states. As in [13], [16], adding a bilingual state extends the parameter set from [*c*, *s*, *a*] to [*c_xz_*, *c_yz_*, *c_zx_*, *c_zy_*, *s*, *a*].

In this paper, we apply the principles of population genetics [26], [27] to language, and combine the simulation and mathematical approaches to study diffusion. We borrow *the Price equation* [28] from evolutionary biology to identify selective pressures on diffusion. Though originally proposed using biological terms, this equation is applicable to any group entity that undergoes transmission in a socio-cultural environment [29]–[31], and involves components that indicate selective pressures at the population level. In addition, this equation relies upon average performance to identify selective pressures, which partials out the influence of initial conditions. Furthermore, compared with Markov chains, this equation needs fewer parameters, which can be estimated from few empirical data. Apart from this equation, we also implement a multi-agent model that follows *the Pólya urn dynamics* from contagion research [32], [33]. This model simulates production, perception, and update of variants during linguistic interactions, and can be easily coordinated with the Price equation.

Empirical studies in historical linguistics and sociolinguistics have shown that linguistic, individual learning and socio-cultural factors could all affect diffusion [8], [10], [34], [35]. In this paper, we focus on some of these factors (e.g., variant prestige, transmission error, individual influence and preference, and social structure), and analyze whether they are selective pressures on diffusion and how non-selective factors modulate the effect of selective pressures.

## Methods

### Price Equation

Biomathematics literature contains several mathematical models of evolution via natural selection, among which the most well-known ones are: (a) *the replicator dynamics* [36], used in the context of evolutionary game theory to study frequency dependent selection; and (b) *the quasi-species model* [37], applicable to processes with constant type-dependent fitness and directed mutations. A third member of this family is the *Price equation* [28], [38], which is mathematically similar to the previous two (see [30]), but has a slightly different conceptual background.

The Price equation is a general description of evolutionary change, applying to any mode of transmission, including genetics, learning, and culture [30], [39]. It describes the changing rate of (the population average of) some quantitative character in a population that undergoes evolution via (possibly non-faithful) replication and natural selection. A special case thereof is the proportion of a certain type in the entire population, which is the character primarily studied by the other two models abovementioned.

In the discrete-time version, the Price equation takes the form as in (2):
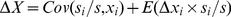
(2)Here, *X* is the population average of the quantitative trait to be studied, and this difference equation denotes the time evolution of this trait. The population is assumed to be divided into *sub-populations* (single individuals or more coarse-grained aggregate objects). Term *s* refers to the average fitness of the population, and *x_i_*, *Δx_i_* and *s_i_* respectively denote the average value of *x*, the difference of this value between subsequent generations, and the average fitness of the *i*th sub-population.

The right-hand side of the equation consists of two terms: a covariance and an expectation. The *covariance* measures the statistical association between fitness and trait value. It captures evolutionary changes due to selection between sub-populations; the stronger the selection for *x*, the stronger the covariance between *x* and fitness. The *expectation* is a fitness-weighted measure of the change in trait value between ancestor and descendant. It tracks changes occurring in sub-populations. If sub-populations are single individuals, the expectation captures unfaithful replication due to mutation or transmission errors; and if sub-populations are more coarse-grained, the covariance captures between-group selection, and the expectation captures both transmission errors and within-group selection.

It is important to note the apparently tautological nature of the Price equation. This nature makes it suitable for describing any dynamic process involving populations at different time points. If there is a complete specification of a dynamic process (say, by means of a Markov chain), the description, by means of the Price equation, of the same process will logically follow the specification. In other words, the Price equation description might be equivalent to the complete dynamic specification, or even contain less information. However, it does not mean that this equation is an alternative to Markov chains or similar specifications of dynamic systems; rather, this equation is a conceptual means. Applying this equation requires clarifying what relations between the stages of the involved population can be considered as *replication* (Price himself did not use this term, but Dawkins' usage of the term [40] is precisely what Price's theory is about). It then provides a clear separation in the population between those changes due to *selection* and those due to other sources. Some scholars criticize Price's approach precisely because the Price equation does not add ant new information to an existing specification of a dynamic process (see for instance [41]), but these critiques do not affect the value of this equation as a conceptual means.

The Price equation can predict the evolution of trait *X* at the population level, *provided the dynamics within sub-populations is well-understood*. It has proven useful especially in clarifying the concept of group selection, since it gives a precise description of the interplay between inter- and intra-group selective forces [39], [42]. To our knowledge, most applications use this equation as *an analytical tool* to derive the dynamic behavior of an aggregate system from the dynamic properties of its components. In this paper, we present another application of this equation, namely as *an empirical tool*. The right-hand side of this equation divides the population-level dynamics into inter- and intra-group selections, plus unfaithful replication. In systems that intra-group selection can be neglected, this corresponds to a division between selection and unfaithful replication. In this way, the Price equation decomposes an empirically observed dynamic process into components respectively tracing selection and unfaithful replication, with the purpose of better understanding the nature of this process.

This paper studies simulations of rather simple dynamic processes inspired by certain features of diffusion. Due to a complex population structure, it is nevertheless not possible (or at least not practical) to give an analytical treatment. However, the Price equation, used in a *top-down* fashion as described above, serves as a tool to measure the selection strength in different scenarios, thus enabling a deeper understanding of the macroscopic properties of the studied processes. As a pilot study adopting this new methodology, we envision practical applications of this approach to non-simulation data from biological and/or cultural evolutions.

### Pólya-urn Model

This model, first designed to study contagion, serves as a suitable model studying diffusion within a population. A Pólya-urn is an urn containing a number of red and green balls; at each time step, a ball is drawn randomly from the urn, and returned to it together with a number of balls of the same or different color. This process is then iterated.

Our model, inspired from this prototypical one, consists of *N* agents (individuals), each denoted by an urn. An urn is initiated with *V* tokens, each belonging to a particular type (*v_1_*, *v_2_*, … *v_V_*) and having a quantifiable *feature x_i_* (all feature values form *F*). At a time step, an interaction occurs between two or more individuals, where a token *v_i_* is drawn randomly from an urn (speaker), and *p_i_* (*prestige* of *v_i_*, all prestige values form *P*) tokens of the same type are added to the speaker itself or other urn(s) (hearer(s)). Here, token drawing corresponds to *production*, and token adding *perception and knowledge update*. Such drawing and adding processes repeat themselves, causing variant type distributions in each urn and the whole population to change over time. *Unfaithful replication* may occur if an added token has a different type from the drawn one.

**Figure 1:**
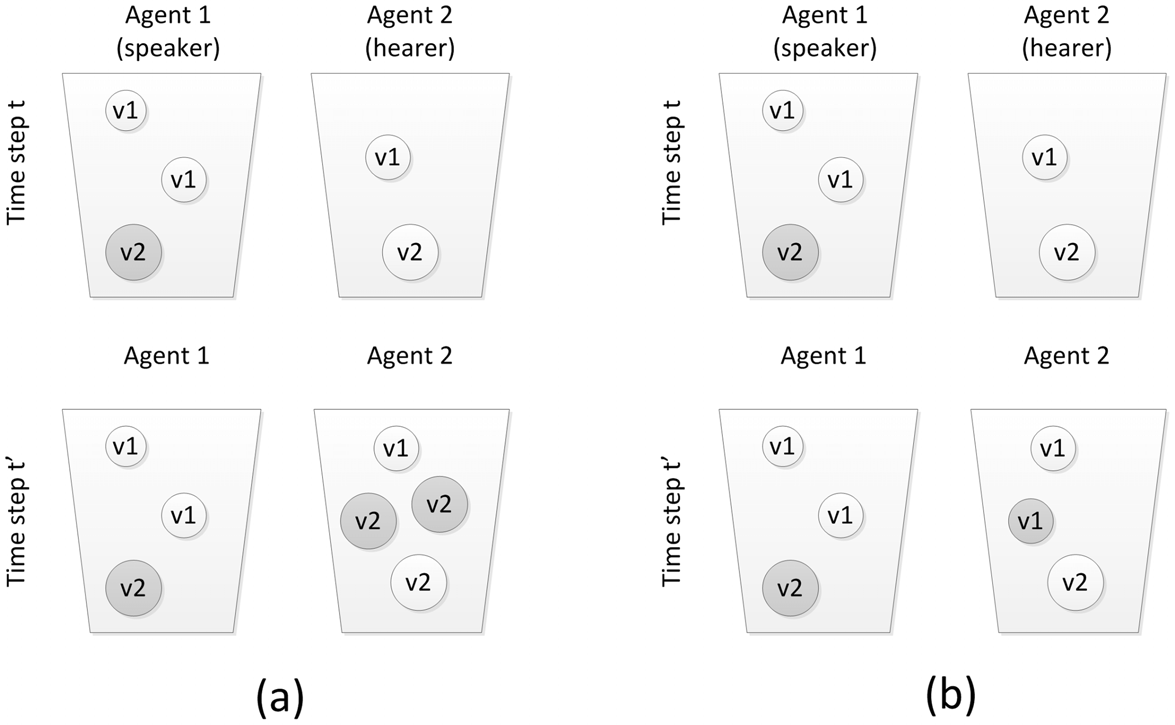
The Pólya-urn dynamics (*N* = 2, *V* = 2, *F* = {1.0, 2.0}). Example (a) involves variant prestige (*P* = {1, 2}) but no unfaithful replication. At time *t*, a token of *v*_2_ is produced by the speaker, due to its prestige (2), two tokens of *v*_2_ are added to the hearer, which forms the state at time *t′*. Example (b) involves unfaithful replication but no variant prestige (*P* = {1, 1}). At *t*, a *v*_2_ is produced, due to unfaithful replication, a *v*_1_ is added, which forms the state at *t′*.

This Pólya-urn model is combinable with the Price equation. We give two examples (see Figure 1) of calculating the Price equation based on this model, under a simple setting: *N* = 2, *V* = 2, *F* = {1.0, 2.0}, i.e., the population has only two agents who exchange only two types of variants. During interactions, only hearers update their urns.

There are two ways to calculate the Price equation, respectively based on two quantifiable features, both reflecting change in the variant type distribution in the population. The first way concerns variant feature *x_i_*, and calculates change in the average feature value *ΔX* between time steps. As in Figure 1(a), the numbers of variants before (*w_i_*) and after (*w′_i_*) the interaction are:
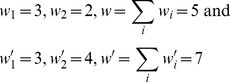
(3)


The relative frequencies (*q_i_*) and fitness (*s_i_*) of variants are
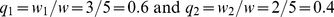
(4)

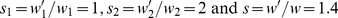
(5)


Then, the covariance is
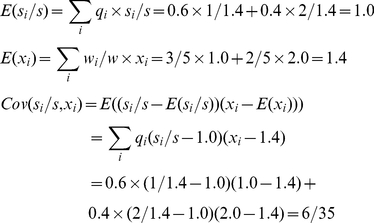
(6)


With no unfaithful replication, offspring variants are identical to their parents, so there is no feature discrepancy, *Δx_i_* = 0.0. Then, the expectation is 0.0. In sum, the right-hand side of the Price equation returns 6/35.

Meanwhile, *ΔX* can be calculated based on the expectations of *x_i_*:
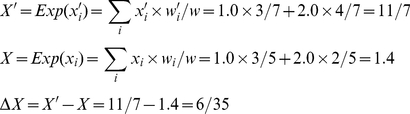
(7)


Both sides return the same value, illustrating the tautology of the Price equation.

As in Figure 1(b), *w* *_1_*, *w_2_*, and *w* are the same as in (3), and *q_1_* and *q_2_* the same as in (4). Here, we need to track which parent type produces the mutated offspring, and calculate the contribution of both faithfully and unfaithfully replicated tokens to the feature value. To this purpose, we define *n_i→j_* as the number of *v_i_* changing to *v_j_*. In this example, *n*_1→1_ = 3, *n*_1→2_ = 0, *n*_2→1_ = 1, *n*_2→2_ = 2. Then
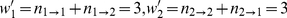
(8)

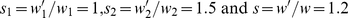
(9)


The covariance is:
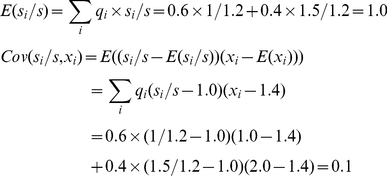
(10)


The feature discrepancies are:
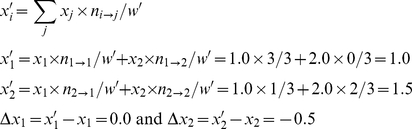
(11)


The expectation is:
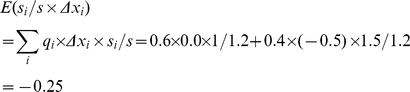
(12)


In sum, the right-hand side returns −0.15. Meanwhile, *ΔX* is:
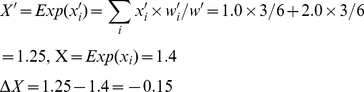
(13)


This calculation also returns −0.15.

In Text S1, we illustrate the second way of calculation, also based on the examples in Figure 1.

These examples show that the Price equation can accurately trace the evolutionary change in the average value of a quantifiable feature in a population. The two ways of calculation identify selective pressures from different angles. *ΔX* in the first way of calculation is determined primarily by the covariance that traces changes in the fitness ratio, whereas the expectation is zero except for unfaithful replication. Then, any factor causing the covariance to be consistently positive or negative can be identified as a selective pressure. However, in the second way, any factor causing the expectation to be consistently positive or negative is a selective pressure.

In our Pólya-urn model, terms “feature”, “prestige”, and “unfaithful replication” have their counterparts in the context of diffusion. Different types of linguistic variants possess *feature values*. Due to certain characteristics, some type of variants can be adopted and used with a higher probability than other(s). Then, this type of variants is said to have a higher *prestige value*. For example, in [6], borrowed lexical variants tend to have higher prestige values than existing ones that encode identical meanings, since the former are more salient to hearers than the latter. Other characteristics, such as the ease in perception or production, may also cause variants to have different prestige values [43]. As in Cantonese, more easily produced pronoun variants ([o5] “I”, [lei5] “you”, and [heoi5] “she/he/it”, numbers indicate tones) have higher prestige values than existing forms ([ngo5], [nei5], and [keoi5]), since the nasals and voiceless plosive in existing forms are more difficult to produce to normal speakers. Finally, during cultural transmission, unfaithful replication usually corresponds to *transmission error*.

Apart from these terms, this Pólya-urn model can also incorporate other individual learning and socio-cultural factors. For example, during diffusion, speakers, hearers, or both, can innovate or have identical or different preferences for variants. Speakers may prefer easily produced variants, as in the case of Cantonese pronouns, whereas hearers may prefer easily perceived or salient ones [4], [44]. Such *individual preference* can be addressed by clarifying situations where only speakers or hearers update their urns. In addition, in a human community, individuals having higher social, political or economic status are more influential than ordinary people [45], [46]. Such *individual influence* can be addressed by defining either a non-uniform distribution of individuals' influences (determining the number of hearers for each agent) or a non-uniform distribution of individuals' popularities (determining the probabilities for agents to participate in interactions). Finally, social connections among individuals can also restrict participants of interactions, thus affecting diffusion. This can be addressed using different types of *social structure*.

## Results

For the sake of simplicity, we only consider two variant types (*V* = 2), and arbitrarily set their feature values as *F* = {1.0, 2.0}. In this case, variant features do not affect the covariance and expectation, since they are cancelled out in the calculation. In cases of multiple types of variants, unless certain types of variants have extremely high or low feature values, variant features will not greatly affect the covariance and expectation. In reality, feature values can denote any quantifiable characteristics of variants, such as vowel length, consonant voicing onset time, lexical item recalling rate, and so on. We set up a 100-agent population and 2000 interactions among these agents (20 interactions per agent) (in the later simulations, the number of interactions can be extended to 5000), and conduct simulations in the following three conditions:

1. Variant prestige with and without transmission error;
2. Individual influence with and without variant prestige;
3. Individual preference and social structure with and without variant prestige;

In each condition, 100 simulations are conducted. In a simulation, we calculate the Price equation at 20 sampling points evenly distributed along 2000 interactions (in simulations having 5000 interactions, 50 sampling points are selected for calculation). Since the Price equation traces only *changes* of variant types, we also measure *Prop* at each sampling point as in (14):
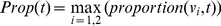
(14)


The average *Prop* over 100 simulations helps evaluate the conclusions drawn from the Price equation. The type of variants having a higher proportion value is referred to as *the majority type*. In Text S2, we show the pseudo codes of the Pólya-urn model and the calculation of the Price equation.

### Variant Prestige with and without Transmission Error

Variant prestige encompasses intrinsic properties of variants, *not* of individuals who carry variants. High prestige value makes certain type of variants more likely to be adopted by individuals. In the simulations of this section, each interaction takes place between two randomly chosen agents, and only hearers update their urns. Variant prestige is introduced via *p_i_*. In conditions with variant prestige, *P* = {1, 2} (conditions *P* = {1, 2} and *P* = {2, 1} are conceptually the same, except that the dominant variant types are different); in those without, *P* = {1, 1}. If *p_i_* = 2, two (instead of one) tokens of the same type are added to the hearer's urn, modeling the enhanced adoption of variants with higher prestige values. Transmission error is introduced via *c* = 0.02, denoting the probability for an added token to become a *mutant* (a token of the other type).

**Figure 2:**
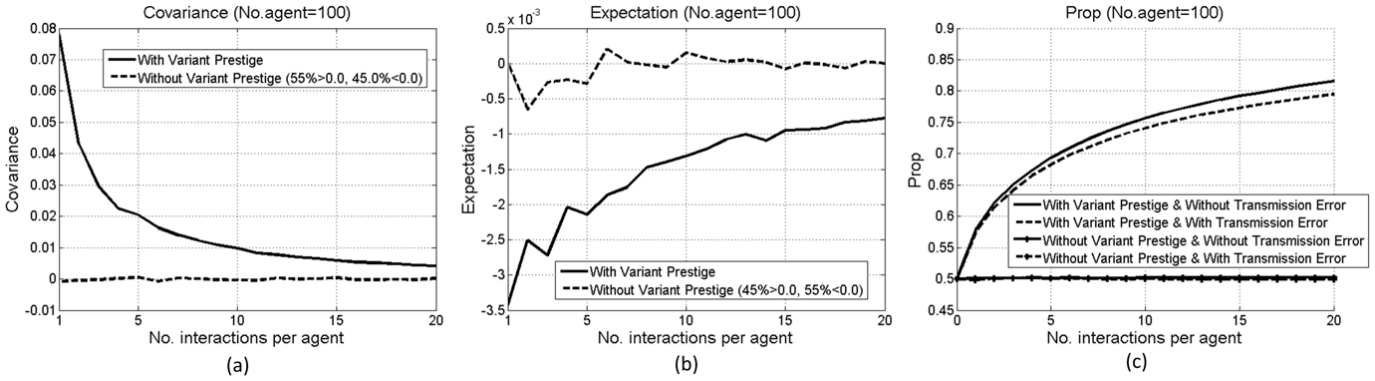
Results of variant prestige and transmission error: (a) covariance without transmission error; (b) expectation with transmission error; (c) *Prop*. Each line is averaged over 100 simulations. Percentage values in the legends denote the proportions of the covariance or expectation above, below or equal to 0.0.

Figures 2(a) and 2(b) show the covariance without transmission error and the expectation with transmission error. With variant prestige, the covariance is consistently positive; otherwise, it fluctuates around 0.0. The gradual decrease in the covariance is due to the increase in the total number of variants, which reduces the effect of a small number of changed variants in each interaction. The consistent positivity of the covariance indicates that variant prestige is a selective pressure on diffusion. Meanwhile, with variant prestige, the expectation is consistently negative; otherwise, it fluctuates around 0.0. This indicates that transmission error reduces the selective pressure of variant prestige, but due to the low error rate, this effect is smaller than that of variant prestige.

Figure 2(c) shows *Prop* in these conditions. With variant prestige, *v_2_*, having a higher prestige value, becomes the majority type, and its *Prop* gradually reaches a high level (above 0.8) (*Prop* never reaches 1.0, since the tokens of *v_1_* are not removed. When they are chosen for production, new tokens of *v_1_* will be added); otherwise, either type can be the majority type in different simulations, and *Prop* remains around 0.5. These results confirm the selective pressure of variant prestige. In addition, Figure 2(c) shows *Prop* in conditions with transmission error (dotted lines). With variant prestige, *Prop* with transmission error is lower than that without, indicating that transmission error reduces the selective pressure of variant prestige; otherwise, *Prop* with and without transmission error are similarly low, around 0.5, indicating that transmission error alone fails to significantly affect diffusion. These findings are in accordance with the conclusions drawn from the Price equation.

**Figure 3:**
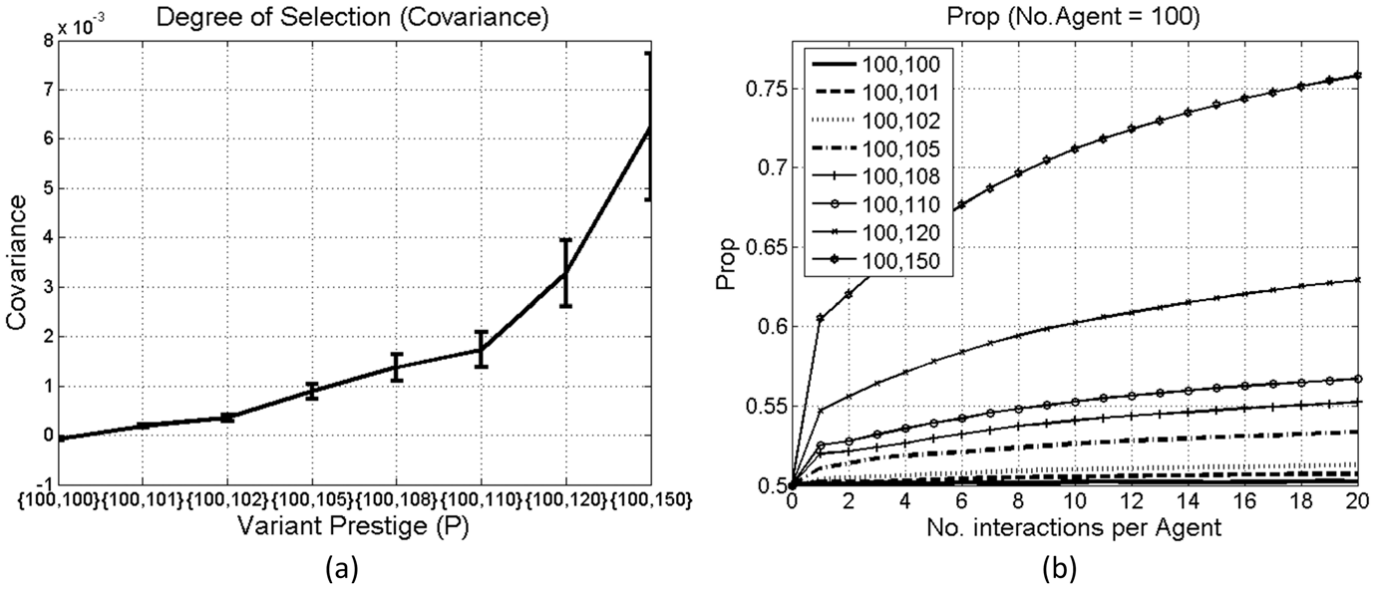
Results under different variant prestige (*P*): (a) average covariance under different *P*; (b) *Prop*. Bars in (a) denote standard errors. Each line in (b) is averaged over 100 simulations.

By adjusting prestige values, we can simulate different degrees of bias for the prestigious type of variants. Adjusting the ratios between the two types of variants is similar to adjusting the functional bias as in [23], [24]. Figure 3 shows *Prop* and average covariance in all the sampling points under different settings of *P*. Once a slight bias for *v_2_* is introduced via *P*, say *P* = {100, 101}, the average covariance will become consistently positive, and the proportion of *v_2_* will be above 0.5. In addition, the average covariance increases along with the increase in the degree of bias for *v_2_*. It means that not only the consistent positivity or negativity of the covariance can reflect selective pressures on diffusion, but the average covariance can also indicate the strength of selection and the degree of diffusion. This is also confirmed by *Prop*.

At the population level, the Price equation and simulation results collectively show that: (a) variant prestige is a selective pressure on diffusion; (b) transmission error can diminish such pressure; and (c) transmission error alone fails to consistently drive diffusion (noting this, we will not consider transmission error in later sections). These conclusions are different from those drawn from an empirical study [45], which finds no effect of variant prestige on diffusion, but the authors of that study admit that their focus is on individual bias and variant prestige is subsumed within that focus.

These conclusions are based on simulations in a finite population and within a limited number of interactions. In Text S3, we prove that these conclusions also hold in a sufficiently large population and an unlimited number of interactions. Meanwhile, single histories of the Pólya-urn dynamics tend to show the reinforcement or lock-in effect [46]. As shown in Figure S1 and discussed in Text S4, such effect cannot affect our conclusions.

### Individual Influence with and without Variant Prestige

Individual influence reflects the fact that members in a community tend to copy the way of certain individuals. Such factor is claimed to be able to enhance the benefit of cultural transmission [47]. In our study, individual influence is discussed in two ways. In the first way, we define a non-uniform distribution of individuals' influences. When an individual speaks, according to its influence, a certain number of other individuals will be randomly chosen as hearers and update their urns according to the token produced by the speaker. Each individual has an equal chance to be chosen as speaker, but the distribution of all individuals' influences follows a *power-law distribution* [49], [50] (inspired from the data in [47], and used in [48]). The power-law distribution has the form 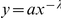, where *x* is the agent index from 1 to *N*, *y* is the influence an agent has, and *a* is a normalizing factor ensuring that the sum of all probabilities is 1.0. The maximum integer smaller than *N*×*y* is the number of hearers influenced by an agent with index *x*. The minimum value of this number is 1. *λ* characterizes different power-law distributions; the higher the *λ*, the more hearers when agents with smaller indices speak.

In the second way, we define a power-law distribution of *individual popularities* (probabilities for individuals to participate in interactions). In this power-law, *y* measures the probability for an individual to interact (as speaker or hearer) with others.

We consider power-law distributions whose *λ* are 0.0, 1.0, 1.5, 2.0, 2.5, and 3.0. *λ* values in many real-world power-law distributions usually fall in this range. If *λ* is 0.0, all agents have the same influence or probability, which resembles the case of random interaction. Values within (0.0 1.0) are excluded, because the influences or probabilities under these values are sensitive to the population size.

**Figure 4:**
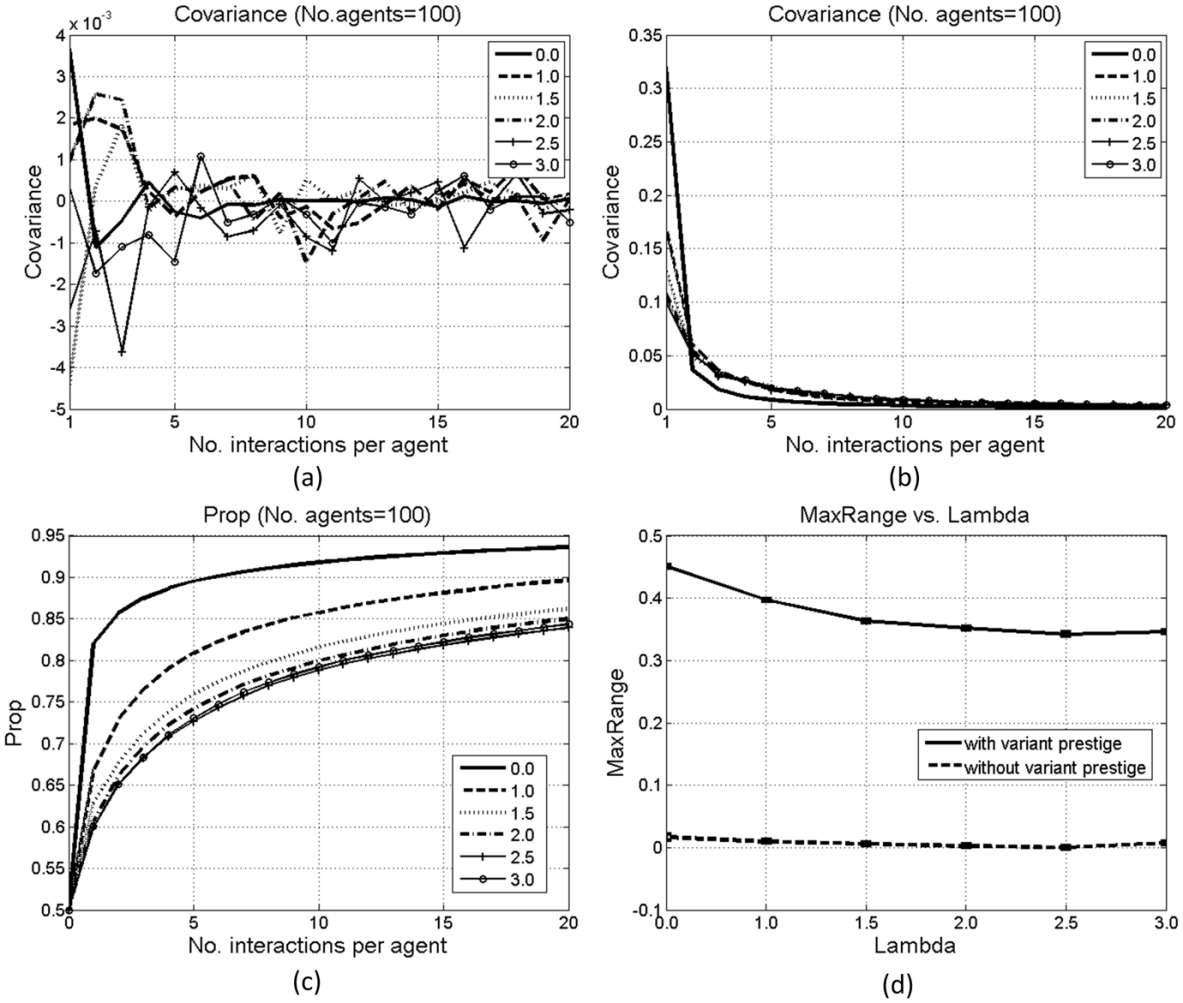
Results with the first type of individual influence: covariance without (a) and with (b) variant prestige; *Prop* with variant prestige (c), and *MaxRange* (d). Each line in (a–c) is averaged over 100 simulations. Bars in (d) denote standard errors.

**Figure 5:**
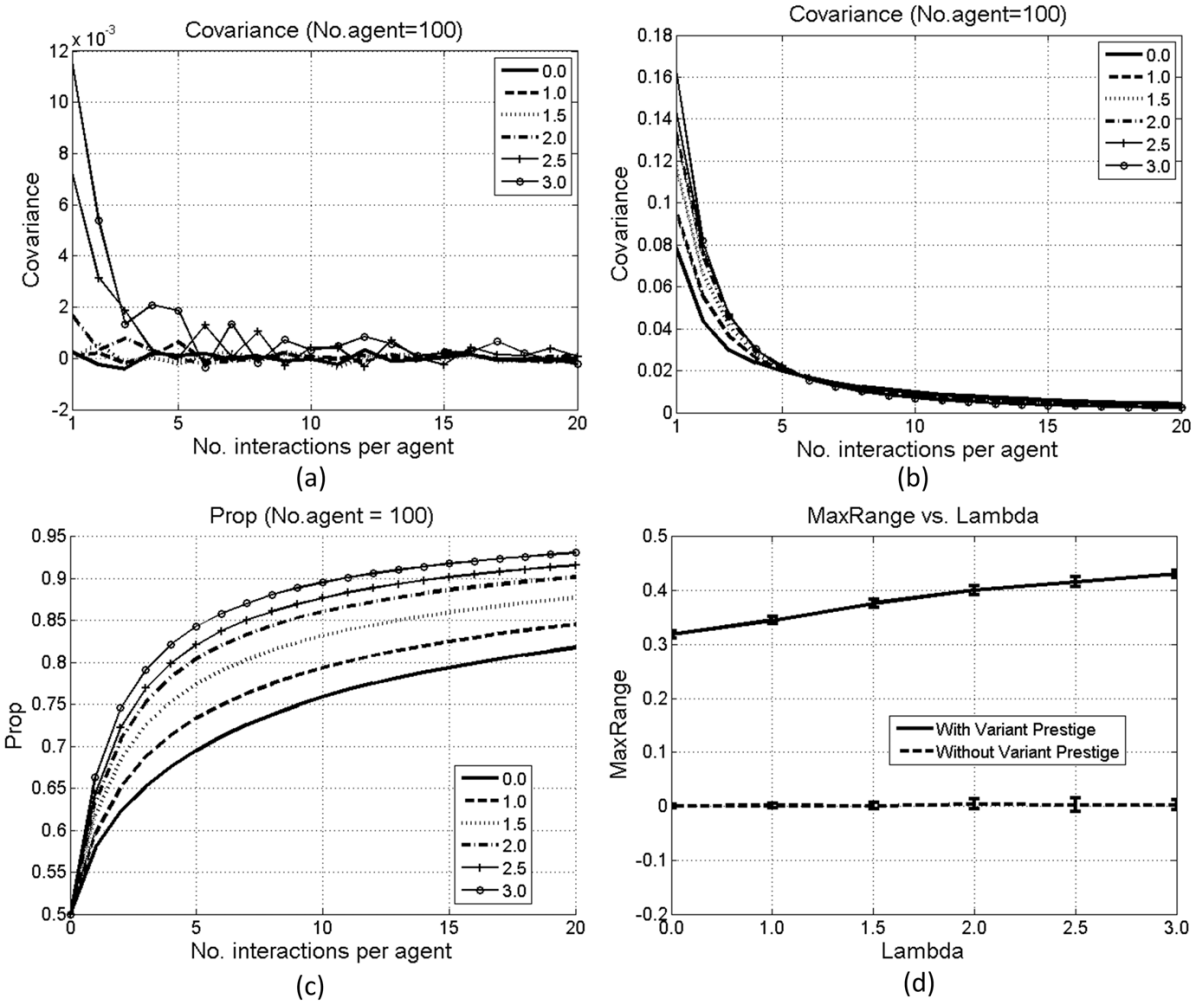
Results with the second type of individual influence: covariance without (a) and with (b) variant prestige, *Prop* with variant prestige (c), and *MaxRange* (d). Each line in (a–c) is averaged over 100 simulations. Bars in (d) denote standard errors.

Figures 4 and 5 show the results under these two types of individual influence. Without variant prestige, both types fail to exert a selective pressure, indicated by the fluctuation of the covariance; otherwise, both can affect diffusion. As shown in Figures 4(c) and 5(c), *λ* and *Prop* are correlated. To illustrate such correlation, we define *MaxRange* as the maximum changing range of *Prop*:
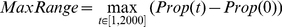
(15)


Figures 4(d) and 5(d) compare *MaxRange* with and without variant prestige. With variant prestige, under the first type of individual influence, there is a negative correlation between *λ* and *MaxRange* (Figure 4(d)). With the increase in *λ*, agents with smaller indices become more influential, who can affect many others, whereas those with bigger indices are less influential, who can only affect 1 or 2 agents. Then, if the influential agents have not developed a clear bias for the prestigious type of variants, their great influence will delay the spread of such bias among others. However, under the second type of individual influence, there is a positive correlation between *λ* and *MaxRange* (Figure 5(d)). With the increase in *λ*, agents with smaller indices will participate in more interactions than others. Then, the proportions of prestigious variants in these agents will have more chances to increase, and the bias for prestigious variants in these agents can get spread to others. Therefore, the diffusion in the whole population is accelerated.

Power-law distribution is omnipresent in social and cognitive domains [51]. We show that in order for the two types of power-law distributed individual influence to significantly affect diffusion, variant prestige is necessary.

### Individual Preference and Social Prestige with and without Variant Prestige

In the above simulations, only hearers update their urns. As discussed before, speakers may also update their urns during interactions. These different ways of introducing new tokens may affect diffusion in a multi-agent population. Meanwhile, a multi-agent population possesses different types of social structure, which could also affect diffusion. Simulations in this section adopt complex networks (treating agents as nodes and interactions as edges) to denote social connections among individuals. We consider 6 types of networks: fully-connected network, star network, scale-free network, small-world network, two-dimensional (2D) lattice, and ring. They characterize many real-world communities. For instance, small-scale societies are usually fully-connected, or have a star-like, centralized structure. Social connections among geographically distributed communities can be denoted by rings or 2D lattices. Large-scale societies generally show small-world and/or scale-free characteristics [47].

Table 1 lists the *average degree* (*AD*, average number of edges per node), *clustering coefficient* (probability for neighbors, directly connected nodes, of a node to be neighbors themselves) and *average shortest path length* (*ASPL*, average smallest number of edges, via which any two nodes in the network can connect to each other) of these networks. Seen from Table 1, from ring to 2D lattice or small-world network, *AD* increases; from 2D lattice to small-world or scale-free network, *ASPL* drops, due to *short-cuts* (edges between non-locally distributed nodes) in small-world network and *hubs* (nodes having many edges connecting others) in scale-free network; and from 2D lattice to scale-free network, and then, to star network, *level of centrality* (*LC*) increases, more nodes become connected to some popular node(s).

**Table 1:** Network characteristics: values are calculated based on 100 nodes.

Network	Average degree	Clustering coefficient	Shortest path length
Fully-connected	99	1.0	1
Star	1.98	0.0	1.98
Scale-free	3.94 (4e-14)	0.14 (0.038)	3.01 (0.071)
Small-world	4	0.17 (0.031)	3.79 (0.086)
2D lattice	4	0.5	12.88
Ring	2	0.0	25.25

Scale-free network is formed by preferential attachment, with average degree around 4; small-world network is formed by rewiring from 2D lattice, with reviewing rate as 0.1. Numbers within brackets are standard deviations of values in scale-free and small-world networks.

In order to gather sufficient data for statistical analysis, we extend the number of interactions to 5000 (50 interactions per agent) and the number of sampling points to 50. There are two sets of simulations: (a) *simulations with speaker's preference*, where only speakers update their urns; and (b) *simulations with hearer's preference*, where only hearers update their urns. In both sets, simulations under the 6 types of network are conducted. In a simulation, only two directly connected agents can interact. Considering that one-speaker-multiple-hearers interactions are common in real societies, we also conduct simulations where all agents directly connected to the speaker can be hearers and update their urns (hearer's preference). These results are shown in Figure S2 and discussed in Text S5.

**Figure 6:**
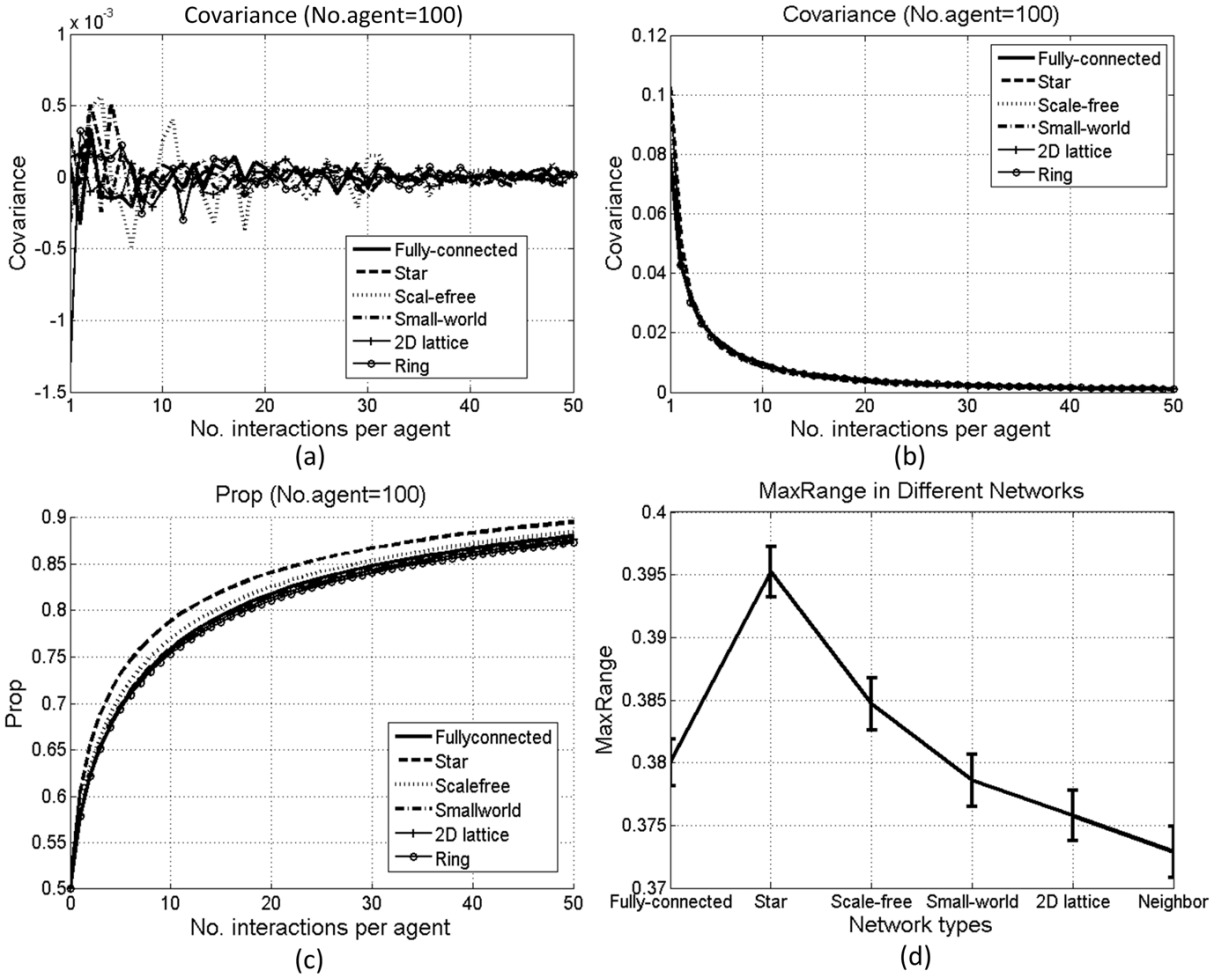
Results with hearer's preference: covariance without (a) and with (b) variant prestige, *Prop* with variant prestige (c), and *MaxRange* with variant prestige (d). Each line in (a–c) is averaged over 100 simulations. Bars in (d) denote standard errors.

Figure 6 shows the simulation results with hearer's preference (results with speaker's preference are similar). Figures 6(a) and 6(b) show that without variant prestige, the covariance fluctuates around 0.0; otherwise, it is consistently positive. Figures 6(c) and 6(d) respectively show *Prop* and *MaxRange* in those networks, given variant prestige.

**Figure 7:**
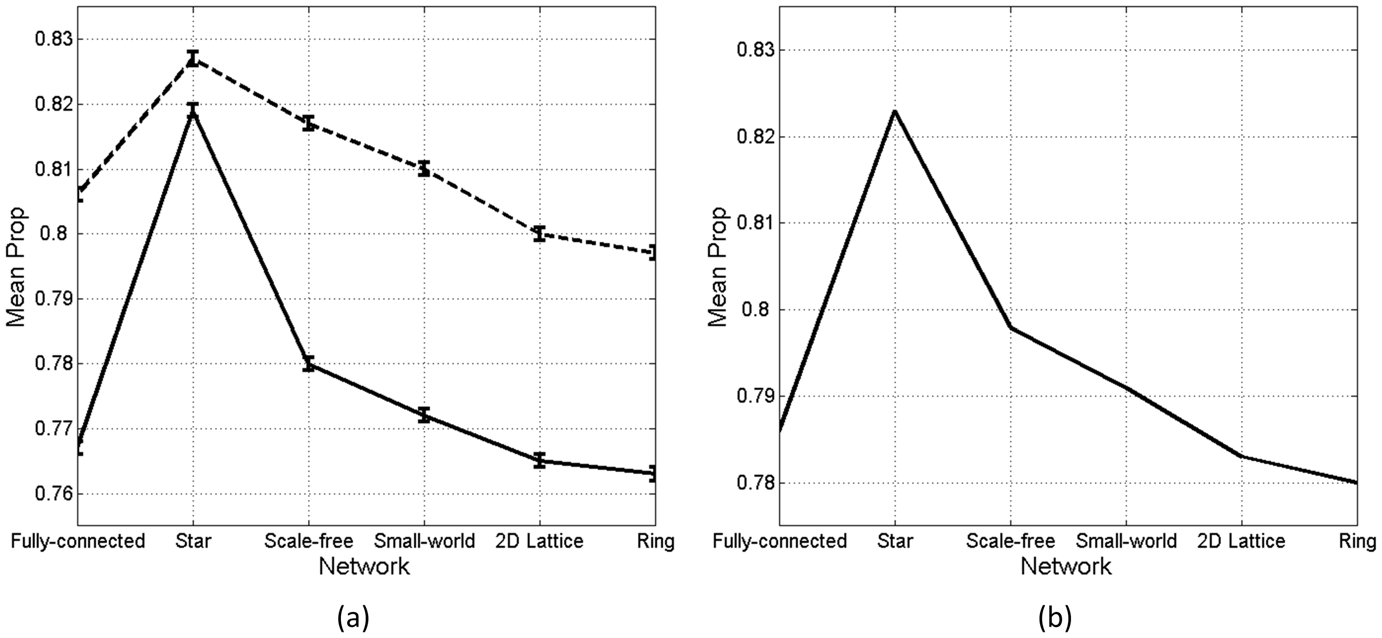
(a) Mean *Prop* with speaker's (solid line) and hearer's preference (dashed line) in different networks. (b) Mean *Prop* over two types of preference in different networks.

Based on *Prop*, we conduct a 2-way analysis of covariance (ANCOVA) (dependent variable: *Prop* over 100 simulations; fixed factors: speaker's/hearer's preference and 6 types of networks; covariate: 50 sampling points along 5000 interactions). This analysis reveals that speaker's or hearer's preference (*F*(1, 61187) = 6905.606, *p*<.001, *η_p_* ^2^ = .101) and networks (*F*(5, 61187) = 1111.425, *p*<.001, *η_p_* ^2^ = .083) have significant main effects on *Prop* (Figure 7). The covariate, number of interactions (sampling points), is significantly related with *Prop* (*F*(1, 61187) = 108285.542, *p*<.001, *η_p_* ^2^ = .639). Instead of ANOVA, using ANCOVA can partial out the influence of the number of interactions.

Figure 7(a) shows that hearer's preference leads to a higher degree of diffusion, compared with speaker's preference. This is evident in not only fully-connected network, which resembles the case of random interactions and excludes network effects, but also other types of networks.

During one interaction, whether the speaker or hearer updates the urn has the same effect on the variant type distribution within these two contacting agents. However, in a situation of multiple agents and iterated interactions, these two types of preference show different effects. Speaker's preference is *self-centered*, disregarding other agents. For example, if an agent has *v_1_* as its majority type, when interacting as the speaker with another agent whose majority type is *v_2_*, it still has a higher chance of choosing a token of *v_1_* and increasing *v_1_*'s proportion by adding more tokens of *v_1_*. In contrast, hearer's preference is *other-centered*, allowing hearer's variant type distribution to be adjusted by other agents. For example, if an agent has *v_1_* as its majority type, when interacting as the hearer with another agent whose majority type is *v_2_*, it will have a higher chance of adding *v_2_* tokens, which will gradually adjust its variant type distribution to be similar to others'. Therefore, given the same number of interactions, hearer's preference is more efficient for diffusion than speaker's preference. In one-speaker-multiple-hearers interactions, the effect of hearer's preference will be further enhanced.

With variant prestige, different types of networks show different degrees of diffusion, as evident in ANCOVA and Figures 6(d) and 7(b). A similar tendency is also shown in Figure S2(d) (except in fully-connected networks). Apart from ANCOVA, we conduct post-hoc T-tests on the mean *Prop* of 100 simulations between different pairs of networks (see Table 2).

**Table 2:** Post-hoc T-test results on the mean *Prop* values of 100 simulations.

Network comparison	Post-hoc T-test result
ring vs. 2D lattice	*t*(198) = −1.206, *p* = 0.229
2D lattice vs. small-world	*t*(198) = −3.239, *p*<0.001 *
small-world vs. scale-free	*t*(198) = −3.884, *p*<0.001 *
scale-free vs. star	*t*(198) = −5.099, *p*<0.001 *
star vs. fully-connected	*t*(198) = 7.482, *p*<0.001 *

“*” marks significant difference.

The different degrees of diffusion in these networks can be ascribed to several structural features of these networks. The first feature is *AD* (average degree). As in Table 1, *AD* is 2 in ring, 4 in 2D lattice. Although in one-speaker-one-hearer interactions, *Prop* between these two networks are not significantly different (see Figure 6(c) and Table 2), in one-speaker-multiple-hearers interactions, the effect of *AD* is explicit (see Figure S3 and Text S5, where we further discuss the effect of *AD* on *Prop*). In addition, the similar results between ring and 2D lattice but different results between 2D lattice and scale-free or small-world network indicate that other structural features are taking effect. And *AD* alone fails to explain why star network, having the lowest average degree (1.98), has the highest *Prop*.

The second feature is short-cuts. From 2D lattice to small-world network, rewiring introduces several short-cuts, and *Prop* in this network is significantly higher than that in 2D lattice (see Table 2, Table S1, and Text S5). However, short-cuts cannot explain why star network, having no such short-cuts, has the highest *Prop*.

The third feature is *LC* (level of centrality). Star network has an extremely centralized structure: there is a hub connecting all other nodes, and this hub participates in all interactions with other nodes. Then, with speaker's preference, the hub has many chances to update its variant type distribution; with hearer's preference, any update of variant type distribution can be quickly spread via the hub to others. Apart from star network, scale-free network, due to preferential attachment, also contains hubs connecting many other nodes, but *LC* in scale-free network is less than that of star network. Accordingly, *Prop* in scale-free network is significantly smaller than that in star network (see Table 2, Table S1, and Text S5). Furthermore, in small-world network, rewiring causes some nodes to have slightly more edges than others, and these nodes will play similar roles as hubs. However, rewiring is less efficient than preferential attachment in forming hubs, so *Prop* in small-world network is significantly smaller than that in scale-free network (see Table 2, Table S1, and Text S5). Finally, lacking centralized structures in other types of networks causes their *Prop* values to be significantly smaller than those of star, scale-free, or small-world networks.

As shown in Table 1, *ASPL* (average shortest path length) reflects a combined effect of *AD*, short-cuts, and *LC*; if a network has a high *AD*, many short-cuts, or a high *LC*, any two nodes in it can be connected via a small number of edges. Since star network has much lower *ASPL* (1.98), it has much higher *Prop*, and then scale-free network (3.01), small-world network (3.79), and 2D lattice (12.88). Since ring has the highest *ASPL* (25.25), its *Prop* is the lowest.

## Discussion

Apart from its successful applications in group selection [39] and altruism [31], the Price equation is introduced in this paper as a new approach for studying language change, especially diffusion. It offers a concise description of evolutionary processes that abstracts away from specific properties of biological evolution [29], [30]. The covariance and expectation in it decompose a dynamic process into a selection and an unfaithful replication component, and quantitative analyses of these components can lead to a better understanding of the effects of various factors on diffusion. Meanwhile, this paper also borrows the Pólya-urn dynamics from contagion studies to simulate diffusion. This dynamics is not language-specific, and simulation results are less dependent on population size and number of variants or interactions. Both features make the findings based on this dynamics also instructive to other socio-cultural phenomena that involve variant transmission.

Based on the Price equation and Pólya-urn dynamics, we identify that variant prestige is a selective pressure that can consistently and explicitly drive the spread of variants with high prestige values in the population. Other factors, including transmission error, individual preference and influence, and social structure, play complementary roles in diffusion, once variant prestige is involved. To be specific, if variants show different prestige values, transmission error can delay diffusion and help preserve less prestigious variants; the two types of individual influence can affect the degree of diffusion in different manners; hearer's preference is more efficient for diffusion than speaker's preference; and structural features, such as average degree, short-cuts and level of centrality, can modulate the degree of diffusion.

These theoretical findings can yield important insights and offer useful guidance on empirical studies of diffusion. As a socio-cultural phenomenon, language evolution proceeds via individual learning and cultural transmission [52]. Our findings suggest that language-external factors in these two aspects have to take effect via language-internal factors, such as variant prestige. Therefore, regarding particular diffusion or other linguistic phenomena, we should not disregard the primary roles of language-internal factors, nor exaggerate the complementary roles of language-external factors. Meanwhile, in empirical studies of diffusion, many observed cases usually occur either as a single history of a particular language or in a small- or medium-scale group of individuals. As discussed in Text S4, genuine selective pressures could be blurred by many factors, such as sampling bias or historical reasons. Therefore, in order to accurately identify selective pressures, we need large-scale sampling, systematical comparison of the available diffusion cases, as well as large-scale, repetitive simulations and statistical analysis as in this study.

Apart from these findings and insights, this paper also exemplifies how computer simulation and mathematical analysis assist each other. The Price equation can quantitatively clarify selective and non-selective factors, but purely from mathematics, it is difficult to evaluate how these factors affect each other during diffusion. Such question can be nicely assessed by simulations under particular settings. Such a combined approach is also instructive to study other language evolution phenomena.

Finally, we can envisage some promising future work from the current study. For instance, heterogeneity (e.g., different individuals have different prestige values on the same type of variants) may cause linguistic diversity or coexistence of multiple types of variants. Simulating contradictory speaker's and hearer's preferences for variants may further reveal the diffusion efficiency of these ways of introducing variants, especially in one-speaker-multiple-hearer interactions. And various forms of cultural transmission among individuals of the same or different generations may also modulate the degree of diffusion. All these can help better elucidate particular diffusion cases in real languages.

## Supporting Information

Text S1 **The second way of calculating the Price equation.**
(DOC)

Text S2 **Pseudo code of the Pólya-urn model.**
(DOC)

Text S3 **The Price equation in continuous time and large population.**
(DOC)

Text S4 **Reinforcement or lock-in effect in Pólya-urn dynamics.**
(DOC)

Text S5 **One-speaker-multiple-hearers interactions with hearer's preference.**
(DOC)

Figure S1 (a) Variant type distribution in each of 100 agents after 2000 interactions in a simulation without variant prestige. (b) Variant type distribution in each of 100 agents after 2000 interactions in a simulation with variant prestige. X axis is agent index, and Y axis is distribution of *v_1_* and *v_2_*. Each bar is divided into two parts: grey part denotes the proportion of *v_1_*, and white part the proportion of *v_2_*. Solid lines mark mean *Prop*. “Above” and “Below” count the number of agents whose *Prop* values are above or below the mean value. (c) Comparison of *Prop* values in simulations with (right column) and without (left column) variant prestige. (d) Mean proportion of *v_2_* across all agents in a particular run without variant prestige (the number of agents is 100). (e) Distribution of proportions of *v_2_* in all 100 agents of the same run. (f) Correlation between the sample standard deviation of proportion of *v_2_* after 50000 interactions and the population size (100 runs under each population size).
(TIF)

Figure S2 **Results of one-speaker-multiple-hearers interactions and hearer's preference: covariance without (a) and with (b) variant prestige,** ***Prop*** **with variant prestige (c), and** ***MaxRange*** **with variant prestige (d).** Each line in (a–c) is averaged over 100 simulations. Bars in (d) denote standard errors.
(TIF)

Figure S3 (a) *Prop* in lattices with different *AD*. (b) Mean *Prop* in lattices with different *AD*. Each line in (a) is averaged over 100 simulations. Bars in (b) denote standard errors.
(TIF)

Table S1 **Post-hoc T-test results on the mean** ***Prop*** **of 100 simulations with one-speaker-multiple-hearers interactions.** “*” marks significant difference.
(DOC)
