# Supplementary material for: Studying Language Change Using Price Equation and Pólya-urn Dynamics
Source: PLoS One. 2012 Mar 12;7(3):e33171. doi: 10.1371/journal.pone.0033171 (PMC3299756; doi:10.1371/journal.pone.0033171)
Supplement: Text S1 — The second way of calculating the Price equation. (DOC) [file pone.0033171.s001.doc]

The Second Way of Calculating the Price Equation

This way of calculation focuses on *individual feature* (*z_i_*, distinct from the first way of calculation), and calculates change in average individual feature *∆Z* between time steps. *z_i_* of agent *i* is contributed by *x_i_* of variants in agent *i*, so *∆Z* indirectly reflects the change in distribution of variants in the population. Since variants produced by a speaker have offspring in other agent(s), those offspring also contribute to the speaker’s feature and fitness. Then, the expectation, tracing feature discrepancy, becomes non-zero, even without unfaithful replication.

Following Figure 1(a), *z_i_* is calculated as below, where *n_i_* records the number of tokens of variant type *i* in a particular agent:

$\begin{matrix} z_{1}=\sum_{i} x_{i}\times n_{i}/\sum_{i} n_{i}=(1.0\times2+2.0\times1)/(2+1)=4/3 \\ z_{2}=(1.0\times1+2.0\times1)/(1+1)=1.5 \end{matrix}$ (S1.1)

*w_i_* records the number of variants (regardless of types) in agent *i*, and *q_i_* the relative frequencies of variants in each agent. In this example,

$\begin{matrix} w_{1}=3,\text{ }w_{2}=2\text{, and }w=\sum_{i} w_{i}=5 \\ q_{1}=w_{1}/w=3/5=0.6\text{ and }q_{2}=2/5=0.4 \end{matrix}$ (S1.2)

*s_i_* records the fitness of agent *i*, which is calculated as the proportion of offspring variants that agent *i* contributes after the interaction. In this example, 2 *v_2_* in the hearer are the offspring of *v_2_* in the speaker. Therefore,

$\begin{matrix} w'_{1}=5\text{ (2 }v_{1}\text{ and 1 }v_{2}\text{ in agent 1, and 2 }v_{2}\text{ in agent 2), }w'_{2}=2\text{, and }w'=\sum_{i} w'_{i}=7 \\ s_{1}=w'_{1}/w_{1}=5/3\text{ , }s_{2}=w'_{2}/w_{2}=2/2=1,\text{ and }s=w'/w=7/5 \end{matrix}$(S1.3)

The covariance is:

$\begin{matrix} E(s_{i}/s)=\sum_{i} q_{i}\times s_{i}/s=0.6\times5/3\div7/5+0.4\times1\div7/5=1.0 \\ E(z_{i})=\sum_{i} q_{i}\times z_{i}=0.6\times4/3+0.4\times1.5=1.4 \\ Cov(s_{i}/s,z_{i})=E((s_{i}/s-E(s_{i}/s))(z_{i}-E(z_{i})))=\sum_{i} q_{i}(s_{i}/s-1.0)(z_{i}-1.4) \\ \text{ }=0.6\times(25/21-1.0)(4/3-1.4)+0.4\times(5/7-1.0)(1.5-1.4) \\ \text{ }=-2/105 \end{matrix}$ (S1.4)

*z’_i_* is calculated based on variants in agent *i* and their offspring elsewhere:

$\begin{matrix} z'_{1}=\sum_{i} x_{i}\times n'_{i}/\sum_{i} n_{i}=(1.0\times2+2.0\times3\text{ (including 2 }v_{2}\text{ in agent 2}))/(2+3)=1.6 \\ z'_{2}=z_{2}=1.5 \end{matrix}$(S1.5)

The expectation is:

$\begin{matrix} \Delta z_{1}=z'_{1}-z_{1}=1.6-4/3=4/15\text{ and }\Delta z_{2}=z'_{2}-z_{2}=0 \\ E(\Delta z_{i}\times s_{i}/s)=\sum_{i} q_{i}\times\Delta z_{i}\times s_{i}/s=0.6\times4/15\times25/21+0.4\times0\times5/7 \\ \text{ }=4/21 \end{matrix}$ (S1.6)

In sum, the right-hand side returns 6/35. Meanwhile, *∆Z* is calculated:

$\begin{matrix} \Delta Z=Exp(z'_{i})-Exp(z_{i})=\sum_{i} x_{i}\times n'_{i}/\sum_{i} n'_{i}-\sum_{i} x_{i}\times n_{i}/\sum_{i} n_{i} \\ \text{ }=(3\times1.0+4\times2.0)/(3+4)-(3\times1.0+2\times2.0)/(3+2) \\ \text{ }=6/35 \end{matrix}$ (S1.7)

This calculation returns the same value as the right-hand side.

In Figure 1(b), after the interaction, 2 *v_1_* and 1 *v_2_* in agent 1 become 3 *v_1_* (1 *v_1_* in agent 2 is unfaithfully replicated from a *v_2_* in agent 1) and 2 *v_2_*, and 1 *v_1_* and 1 *v_2_* in agent 2 do not change. Then,

$\begin{matrix} w'_{1}=4,\text{ }w'_{2}=2\text{, and }w'=\sum_{i} w'_{i}=6 \\ s_{1}=w'_{1}/w_{1}=4/3\text{ , }s_{2}=w'_{2}/w_{2}=2/2=1,\text{ and }s=w'/w=6/5 \end{matrix}$ (S1.8)

After the interaction,

$\begin{matrix} z'_{1}=\sum_{i} x_{i}\times n'_{i}/\sum_{i} n_{i}=(1.0\times3\text{ (including 1 }v_{1}\text{ in agent 2)}+2.0\times1)/(3+1)=5/4 \\ z'_{2}=z_{2}=1.5 \end{matrix}$(S1.9)

The covariance and expectation are:

$\begin{matrix} Cov(s_{i}/s,z_{i})=E((s_{i}/s-E(s_{i}/s))(z_{i}-E(z_{i})))=\sum_{i} q_{i}(s_{i}/s-1.0)(z_{i}-1.4) \\ \text{ }=0.6\times(10/9-1.0)(4/3-1.4)+0.4\times(5/6-1.0)(1.5-1.4) \\ \text{ }=-1/90 \end{matrix}$ (S1.10)

$\begin{matrix} \Delta z_{1}=z'_{1}-z_{1}=5/4-4/3=-1/12\text{ and }\Delta z_{2}=z'_{2}-z_{2}=0 \\ E(\Delta z_{i}\times s_{i}/s)=\sum_{i} q_{i}\times\Delta z_{i}\times s_{i}/s=0.6\times(-1/12)\times10/9+0.4\times0\times5/6 \\ \text{ }=-1/18 \end{matrix}$ (S1.11)

In sum, the right-hand side returns −1/15. Meanwhile, *∆Z* is calculated:

$\begin{matrix} \Delta Z=Exp(z'_{i})-Exp(z_{i}) \\ \text{ }=(4\times1.0+2\times2.0)/(4+2)-(3\times1.0+2\times2.0)/(3+2) \\ \text{ }=-1/15 \end{matrix}$ (S1.12)

This calculation returns the same value.

*∆Z* in this way of calculation is determined primarily by the expectation that takes account of both fitness ratio and feature discrepancy. Feature discrepancy indirectly traces changes in variant type distribution in the population. Any factor causing the expectation to be consistently positive or negative can be identified as a selective pressure.
