# Supplementary material for: Studying Language Change Using Price Equation and Pólya-urn Dynamics
Source: PLoS One. 2012 Mar 12;7(3):e33171. doi: 10.1371/journal.pone.0033171 (PMC3299756; doi:10.1371/journal.pone.0033171)
Supplement: Text S2 — Pseudo code of the Pólya-urn model. (DOC) [file pone.0033171.s002.doc]

Pseudo Code of the Pόlya-urn Model

This model is implemented by C++. The program defines two classes: *CVar* (variants) and *CAg* (interacting agents). CVar has 3 members: *Feature* (variant feature), *ParVar* and *OffVar* (pointing to parent and offspring variants). CAgent has 2 members: *VarList* (list of variants) and *VarListSize* (number of variants).

Main program

int pop_Size=100, var_Type=2, no_Inter=2000; // population size, variant types, no. of interactions;

int sam_fre=10, F[]=[1 2]; // sampling frequency and feature values;

int P[]=[1 2] (or [1 1]); // variant prestige, P=[1 1] for cases without variant prestige;

double c=0.0 (or 0.02); // mutation rate; c=0.02 for cases with transmission error;

CAg Agent[pop_Size]; // 100 agents;

int spInd, heInd; // speaker’s and hearer’s index;

CVar *var=NULL; // var points to a chosen variant;

// initialization;

for (i=0;i<pop_Size;i++) { // each agent has one variant of each type;

Agent[i]->VarListSize=var_Type; Agent[i]->VarList=new CVar [Agent[i]->VarListSize];

for (j=0;j< Agent[i]->VarListSize;j++) {

Agent[i]->VarList[j]->Feature=F[j];

Agent[i]->VarList[j]->ParVar=NULL; Agent[i]->VarList[j]->Off=NULL;

}

}

// interaction;

for (i=0;i<no_Inter;i++) {

do { spInd=(int)(rand(0, pop_Size-1)); heInd=(int)(rand(0, pop_Size-1)); }while(spInd==heInd);

// speaker’s and hearer’s indices are randomly chosen in [0 pop_Size-1] in random interaction

// simulations, according to power-law distribution, PowDist, in individual influence simulations,

// and following the adjacent matrix, NetAdj, in social structure simulations;

var=Agent[spInd]->VarList[(int)(rand(0, Agent[spInd]->VarListSize-1)]; // random selection;

Agent[heInd]->addVar(var, P, c); // hearer adds tokens according to var, P, and c;

if (i%sam_fre==0) calPriceEquation(i, VarOld, F); // calculate Price equation; VarOld stores the

// numbers of different types of variants at the previous sampling point;

}

Function: addVar(var, P, c)

int noNewVar; // record the number of newly added variant tokens;

CVar *newVar=NULL; // points to a newly created variant token;

if (var->Feature==1) noNewVar=P[0];

else noNewVar=P[1]; // decide the number of added tokens based on var’s type;

for (i=0;i<noNewVar;i++) {

newVar=new CVar; // create a new token;

if(rand(0,1.0)<=c) newVar->Feature=var_Type+1-var->Feature; // mutation occurs;

else newVar->Feature=var->Feature; // no mutation;

newVar->ParVar=var; newVar->OffVar=NULL; // newVar is added by var, to trace mutation;

add(VarList, newVar); VarListSize++; // add newVar into VarList;

}

Function: calPriceEquation(numInter, VarOld, F): (based on the first way of calculation)

int VarNew[]=[0 0]; // record the number of different types of variants;

int sumOld=0, sumNew=0; // total numbers of variants in previous and current sampling points;

double q[]=[0.0 0.0]; s[]=[0.0 0.0], s_all=0.0; // relative frequencies and fitness, see equations (4)(5);

double expXi=0.0, expSi_S=0.0, cov=0.0, exp=0.0; // see equation (6);

for (i=0;i<pop_Size;i++) {

for (j=0;j<Agent[i]->VarListSize;j++) {

for (k=0;k<var_Type;k++) {

if (Agent[i]->VarList[j]->Feature==F[k]) VarNew[k]++;

}

}

} // count the number of variants of the same type in all agents;

// calculate q, s and s_all, and expXi and expSi_S as in equations (4)-(6);

for (i=0;i<var_Type;i++) {

sumOld+=VarOld[i]; sumNew+=VarNew[i]; s[i]=VarNew[i]/VarOld[i];

}

s_all=sumNew/sumOld;

for (i=0;i<var_Type;i++) {

q[i]=VarOld[i]/sumOld; expXi+=VarOld[i]/sumOld*F[i]; expSi_S+=q[i]*s[i]/s_all;

}

// calculate cov and exp in the Price equation as in equation (6);

for (i=0;i<var_Type;i++) {

cov+=q[i]*(s[i]/s-expSi_S)*(F[i]-expXi);

}

exp=0.0; // for transmission error, further calculation is needed here, following equations (8)-(12);

fprintf(‘PriceRes.txt’, “%d %f %f”, numInter, cov, exp); // record results into file;

for (i=0;i<var_Type;i++) {

VarOld[i]=VarNew[i];

} // update VarOld with NewOld; VarOld is transferred back to the main program for future usage;
