# Supplementary material for: Studying Language Change Using Price Equation and Pólya-urn Dynamics
Source: PLoS One. 2012 Mar 12;7(3):e33171. doi: 10.1371/journal.pone.0033171 (PMC3299756; doi:10.1371/journal.pone.0033171)
Supplement: Text S3 — The Price equation in continuous time and large population. (DOC) [file pone.0033171.s003.doc]

The Price Equation in Continuous Time and Large Population

The following proof is based on the first way of calculating the Price equation and the continuous version of the Price equation [38]:

$\mathop{X}\limits^{\bullet} =Cov(s_{i},x{}_{i})+E(\mathop{x_{i}}\limits^{\bullet} )$ (S3.1)

Frist, we consider the case with variant prestige but no transmission error. If one time unit consists of *M* steps, the probability for a token to be chosen in each round is 1/*M*. Since each token produces offspring according to its prestige, *s_1_*=1 and *s_2_*=2. Without transmission error, $\mathop{x}\limits^{\bullet} _{i}=0$.

Then,

$\begin{matrix} E(s_{i})=1\times q_{1}+2\times q_{2}=2-q_{1} \\ E(x_{i})=1.0\times q_{1}+2.0\times q_{2}=2-q_{1} \\ Cov(s_{i},x_{i})=E[(s_{i}-E(s_{i}))(x_{i}-E(x_{i}))]=\sum_{i} q_{i}(s_{i}-2+q_{1})(x_{i}-2+q_{1}) \\ \text{ }=q_{1}(q_{1}-1)^{2}+q_{2}q_{1}^{2}=q_{1}((q_{1}-1)^{2}+q_{1}-q_{2}^{2})=q_{1}(1-q_{1}) \end{matrix}$ (S3.2)

Meanwhile,

$X=q_{1}\times1.0+(1-q_{1})\times2.0=2-q_{1},\text{ hence }\mathop{X}\limits^{\bullet} =-\mathop{q_{1}}\limits^{\bullet}$ (S3.3)

Combining both,

$\mathop{q_{1}}\limits^{\bullet} =q_{1}(q_{1}-1)$ (S3.4)

The solution to this differential equation is:

$q_{1}=1/(1+\exp(t+K)),\text{ while }K\text{ is a constant}$ (S3.5)

Given unlimited *t*, *q_1_* converges to 0.0 (and *q_2_* to 1.0), i.e., a high prestige on *v*_2_ helps *v*_2_ diffuse in the population.

Second, we consider the case with variant prestige and transmission error (error rate is *c*). The covariance remains unchanged, but the expectation becomes:

$E(\mathop{x}\limits^{\bullet} )=q_{1}\times c\times(2.0-1.0)+(1-q_{1})\times c\times(1.0-2.0)=(2q_{1}-1)c$ (S3.6)

The Price equation is:

$\mathop{X}\limits^{\bullet} =q_{1}(1-q_{1})+(2q_{1}-1)c$ (S3.7)

Considering$\mathop{X}\limits^{\bullet} =-\mathop{q}\limits^{\bullet} _{1}$,

$\mathop{q_{1}}\limits^{\bullet} =q_{1}(q_{1}-1)+(1-2q_{1})c$ (S3.8)

It is difficult to solve this equation analytically, but there is only one fixed point within [0.0 1.0]:

$q_{1}^{*}=(1+2c-\sqrt{1+4c^{2}})/2$ (S3.9)

Since

$\partial\mathop{q{}_{1}}\limits^{\bullet} /\partial q_{1}|_{q_{1}=q_{1}^{*}}<0$ (S3.10)

This fixed point is an attractor. For *c*=0.02, it is around 0.0196. This shows that due to transmission error, *v*_1_ does not die out completely and its proportion converges to a low and stable value dependent on *c*. This result is in line with the conclusion in the main text.

Finally, we consider the case without variant prestige but with transmission error. Here, *s_1_*=*s_2_*=1. Following the previous calculation,

$\begin{matrix} X=1.0\times q_{1}+2.0\times q_{2}=2-q_{1} \\ E(s_{i})=q_{1}+q_{2}=1 \\ E(x_{i})=X=2-q_{1} \\ Cov(s_{i},x_{i})=E[(s_{i}-E(s_{i}))(x_{i}-E(x_{i}))]=\sum_{i} q_{i}(s_{i}-1)(x_{i}-2+q_{1})=0 \end{matrix}$(S3.11)

The covariance becomes 0, indicating that transmission error exerts no selective pressure. Meanwhile, the expectation is$(2q_{1}-1)c$. Then, the Price equation becomes:

$\mathop{q_{1}}\limits^{\bullet} =(1-2q_{1})c$ (S3.12)

The solution to it is$q_{1}=0.5$, independent of *c*. This shows that given unlimited *t* both types of variants will have roughly the same proportions, which is also consistent with the conclusion in the main text.
