# Supplementary material for: Studying Language Change Using Price Equation and Pólya-urn Dynamics
Source: PLoS One. 2012 Mar 12;7(3):e33171. doi: 10.1371/journal.pone.0033171 (PMC3299756; doi:10.1371/journal.pone.0033171)
Supplement: Text S4 — Reinforcement or lock-in effect in Pólya-urn dynamics. (DOC) [file pone.0033171.s004.doc]

Reinforcement or Lock-in Effect in Pόlya-urn Dynamics

In our Pόlya-urn model, due to particular interacting history, some agents may have more *v_1_* than *v_2_* (or vice versa). More interactions in which these agents are speakers will enhance the bias for *v_1_* (or *v_2_*), and accumulation of such reinforcement in particular runs may affect the distribution of variant types in the population. In [46,47], such effect is referred to as the reinforcement or lock-in effect.

Without variant prestige, the lock-in effect is evident in our simulations. As shown in a particular run (see Figure S1(a)), the proportion of *v_1_* (the grey part) is less than 0.3 in some agents (pointed by black arrows), but around 0.7 in others (pointed by grey arrows), and the *Prop* of the population after 2000 interactions is above the expected value 0.5 (0.53). However, across simulations, the lock-in effect only plays a minor role. As shown in Figure 2(c), without variant prestige, the average proportion of the majority type (either *v_1_* or *v_2_*) across simulations is close to 0.5, indicating that the average proportion of the minority type must be close to 0.5 as well, i.e. the simulations do converge to a value close to the expected one.

With variant prestige, the lock-in effect is even weaker. As shown in a particular run (see Figure S1(b)), some agents (pointed by black arrows) could have a relatively high proportion of *v_1_*, around 0.4, but the high mean *Prop* of the population indicates an explicit bias for *v_2_*. Across simulations, a post-hoc T-test shows that *Prop* values in simulations with and without variant prestige are significantly different (*t*(198) = -82.8945, *p* < 0.001), indicating that the lock-in effect cannot hold back the selective pressure of variant prestige.

Considering the small number of runs in each condition (100), we conduct another 900 runs without variant prestige and 900 runs with variant prestige. Together with the available 200 runs, we compare the *Prop* values after 2000 interactions in these 2000 runs (see Figure S1(c)). Without variant prestige, *Prop* may reach 0.6 or 0.4 in certain runs (pointed by black arrows), but the average *Prop* is close to the expected value 0.5. With variant prestige, however, in all 1000 runs, there is no agent whose number of *v_1_* is bigger than that of *v_2_* after 2000 interactions, and no run has its *Prop* near 0.5 (the minimum is around 0.75, pointed by a grey arrow). These reflect an explicit bias for *v_2_*.

Considering the small number of interactions (2000), we further conduct 100 runs without variant prestige, each involving 50000 interactions. As shown in a particular run (see Figure S1(d)), the proportion of *v_2_* ends up at a value below the expected value. However, across 100 runs, we observe approximately a normal distribution whose mean is close but not identical to 0.5 (see Figure S1(e)). In other words, without variant prestige, the proportion of *v_2_* in the entire population could be a random variable, having 0.5 as its mean but a non-vanishing variance across simulations.

Such variance becomes smaller with the increase in the number of agents (urns). To illustrate this, we start with a 100-agent population and gradually increase the population size with a step of 100 until 2000. In each size, we conduct 100 runs with 50000 interactions each. Figure S1(f) traces the sample standard deviation of the proportion of *v_2_* within all agents after 50000 interactions. The standard deviation approximately follows a power law relation with the population size (note the log-log axes). Accordingly, in the limit of an infinite population, the lock-in effect will disappear.

The lock-in effect shown in the Pόlya-urn dynamics is evident in real diffusion cases. For instance, as in the case of Cantonese pronouns, there are individuals who do not show explicit biases for easily-pronounced, high-prestige forms, partially due to their language learning experiences. Small-scale or biased sampling involving such individuals may blur the effect of genuine selective pressures. To clarify whether factors observed in particular cases are genuine selective pressures, a large-scale, unbiased sampling and a systematic comparison of available cases are necessary.
