# Supplementary material for: Studying Language Change Using Price Equation and Pólya-urn Dynamics
Source: PLoS One. 2012 Mar 12;7(3):e33171. doi: 10.1371/journal.pone.0033171 (PMC3299756; doi:10.1371/journal.pone.0033171)
Supplement: Text S5 — One-speaker-multiple-hearers interactions with hearer's preference. (DOC) [file pone.0033171.s005.doc]

One-Speaker-Multiple-Hearers Interactions with Hearer’s Preference

Figure S2 shows the results of one-speaker-multiple-hearers simulations and hearer’s preference (the results with speaker’s preference are similar). Similar to one-speaker-one-hearer simulations, different networks (except fully-connected network) show different degrees of diffusion. Table S1 summarizes the results of post-hoc T-tests between these networks.

To evaluate the effect of *AD* on *Prop*, we conduct simulations based on lattices with different *AD* (2, 4, 8, 16, 32, and 64), and an ANCOVA (dependent variable: *Prop* over 100 simulations (due to the software limitation of SPSS); fixed factors: different *AD*; covariate: 50 sampling points along 5000 interactions). This analysis reveals a significant main effect of *AD* on *Prop* (*F*(5, 30593) = 1865.642, *p* < .001, *η_p_*^2^ = .234) (Figure S3). The covariate, number of interactions (sampling points), is also significantly related with *AD* (*F*(1, 30593) = 14115.832, *p* < .001, *η_p_*^2^ = .316).
