# Supplementary figures and images for: Studying Language Change Using Price Equation and Pólya-urn Dynamics
Source: PLoS One. 2012 Mar 12;7(3):e33171. doi: 10.1371/journal.pone.0033171 (PMC3299756; doi:10.1371/journal.pone.0033171)

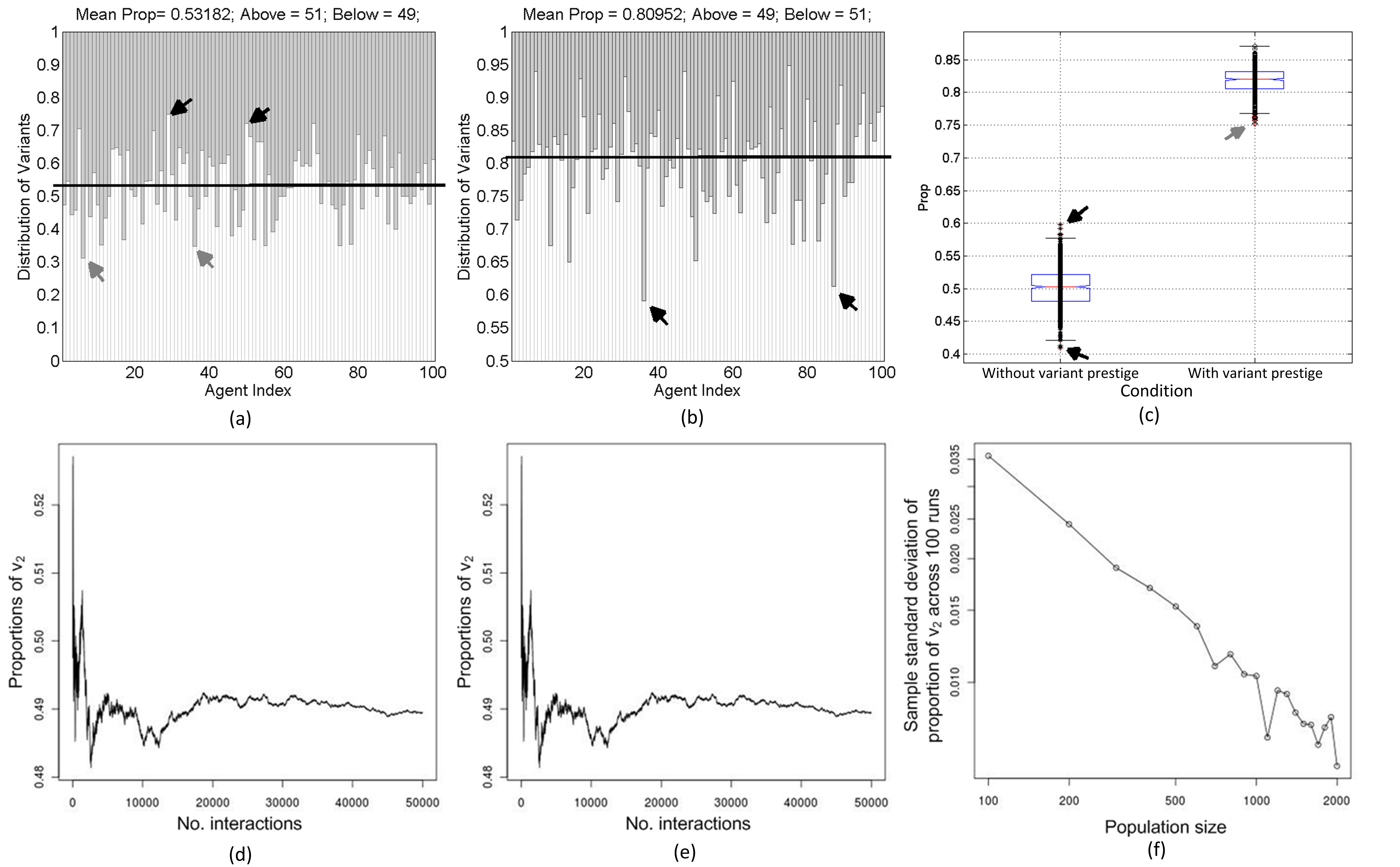

Supplement: Figure S1 — (a) Variant type distribution in each of 100 agents after 2000 interactions in a simulation without variant prestige. (b) Variant type distribution in each of 100 agents after 2000 interactions in a simulation with variant prestige. X axis is agent index, and Y axis is distribution of v1 and v2. Each bar is divided into two parts: grey part denotes the proportion of v1, and white part the proportion of v2. Solid lines mark mean Prop. “Above” and “Below” count the number of agents whose Prop values are above or below the mean value. (c) Comparison of Prop values in simulations with (right column) and without (left column) variant prestige. (d) Mean proportion of v2 across all agents in a particular run without variant prestige (the number of agents is 100). (e) Distribution of proportions of v2 in all 100 agents of the same run. (f) Correlation between the sample standard deviation of proportion of v2 after 50000 interactions and the population size (100 runs under each population size). (TIF) [file pone.0033171.s006.tif]

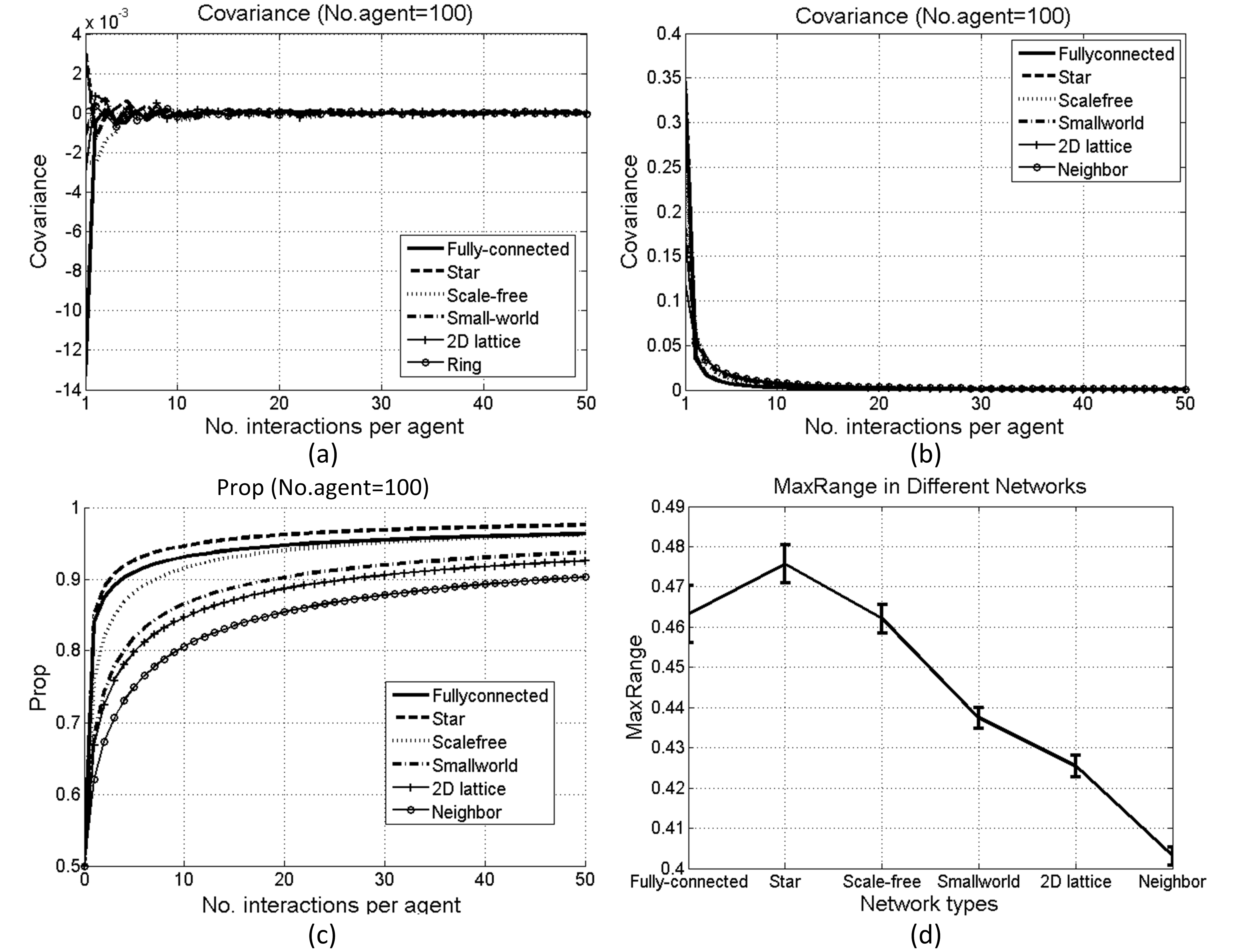

Supplement: Figure S2 — Results of one-speaker-multiple-hearers interactions and hearer's preference: covariance without (a) and with (b) variant prestige, Prop with variant prestige (c), and MaxRange with variant prestige (d). Each line in (a–c) is averaged over 100 simulations. Bars in (d) denote standard errors. (TIF) [file pone.0033171.s007.tif]

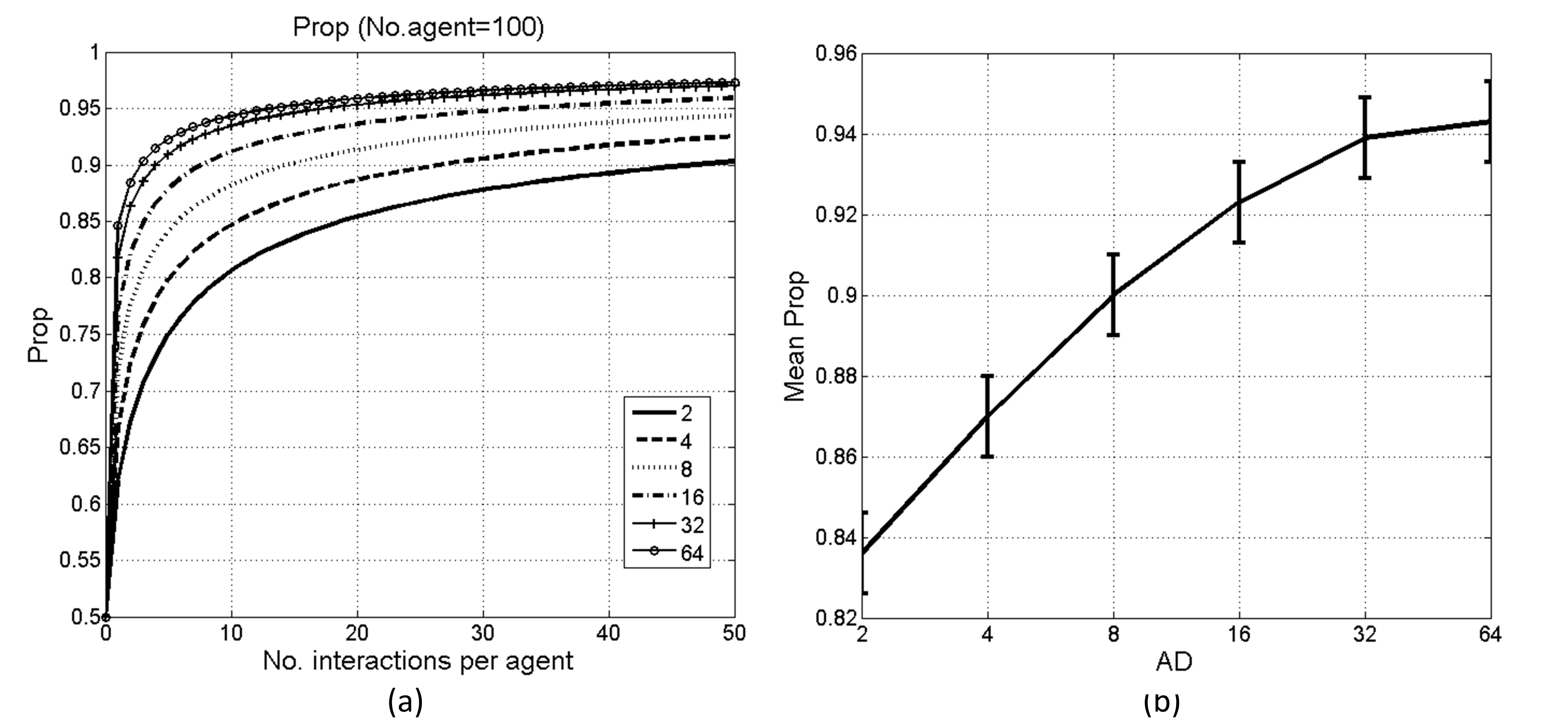

Supplement: Figure S3 — (a) Prop in lattices with different AD. (b) Mean Prop in lattices with different AD. Each line in (a) is averaged over 100 simulations. Bars in (b) denote standard errors. (TIF) [file pone.0033171.s008.tif]
