# Supplementary material for: Studying Language Change Using Price Equation and Pólya-urn Dynamics
Source: PLoS One. 2012 Mar 12;7(3):e33171. doi: 10.1371/journal.pone.0033171 (PMC3299756; doi:10.1371/journal.pone.0033171)
Supplement: Table S1 — Post-hoc T-test results on the mean Prop of 100 simulations with one-speaker-multiple-hearers interactions. “*” marks significant difference. (DOC) [file pone.0033171.s009.doc]

**Table S1.** Post-hoc T-test results on the mean *Prop* of 100 simulations with one-speaker-multiple-hearers interactions. “*” marks significant difference.

| Network comparison | Post-hoc T-test result |
| --- | --- |
| ring vs. 2D lattice | *t*(198) = -9.344, *p* < 0.001 * |
| 2D lattice vs. small-world | *t*(198) = -6.813, *p* < 0.001 * |
| small-world vs. scale-free | *t*(198) = -10.144, *p <* 0.001 *** |
| scale-free vs. star | *t*(198) = -3.185, *p <* 0.001 *** |
